# The effects of whole-body muscle stimulation on body composition and strength parameters: A protocol for systematic review and meta-analysis

**DOI:** 10.1097/MD.0000000000025139

**Published:** 2021-05-07

**Authors:** Luiz Rodrigues-Santana, José Carmelo Adsuar, Hugo Louro, Jorge Pérez-Gómez, Miguel Angel Hernández-Mocholí, Jorge Carlos-Vivas, Rossana Gomez-Campos, Luis Felipe Castelli Correia de Campos

**Affiliations:** a University of Extremadura; b Health, Economy, Motricity and Education; c Research Center in Sport Sciences, Health and Human Development, Vila Real, Portugal; d Universidad Católica del Maule, Talca; e Universidad del Bio Bio, Chillán, Chile.

**Keywords:** body composition, electromyostimulation, fat mass, lean body mass, strength, whole body muscle stimulation

## Abstract

**Background:**

This study will analyze the effect of Whole Body Electromyostimulation (WB-EMS) in strength and body composition outcomes in adult population.

**Methods:**

This study will search the following electronic databases up to July 21, 2020: PubMed, WOS, Scopus, SPORTDiscus y EMBASE. There will be no language limitation. Two authors will independently identify titles/abstracts and full text all potential studies, and will collect data from eligible studies. Additionally, study quality will be assessed by PEDro Scale risk of bias. We will conduct meta-analysis if enough trials are included.

**Results:**

This study will explore the effects of WB-EMS in strength and body composition outcomes.

**Conclusion:**

The findings of this study may summarize the effectiveness of WB-EMS in increasing strength and improving body composition in adult population.

**INPLASY registration number:**

INPLASY202120050

## 1 Introduction

Body composition is 1 of the main indicators of physical health and well-being. In fact, its changes throughout life are related to the risk of mortality.^[1]^ According to the American Council on Exercise,^[2]^ the percentage of healthy fat for adults is up to 24% for men and 31% for women, with higher values being considered excess body fat, which is the main cause of obesity and other metabolic and cardiovascular diseases. In addition, the amount of muscle mass and strength play an important role, since as we age, muscle mass tends to decrease and its loss is directly related to a decrease in functional capacity, a worse quality of life and dependence in the elderly.^[3]^ It is generally known that physical exercise is essential for a healthy life. The American College of Sports and Medicine recommends a regular practice of cardiovascular physical activity of 150 minutes a week with two sessions of muscular resistance training for the main muscle groups, for the improvement and maintenance of cardiorespiratory, skeletal muscle and neuromotor fitness in apparently healthy people.^[4,5]^

High intensity training programs have a positive impact on fat loss and increased muscle mass,^[6]^ being today one of the most used strategies to improve body composition, since this training method has shown results similar to those obtained after applying a traditional continuous training program of moderate intensity, with 40% less duration.^[7]^

Whole body muscle stimulation (WB-EMS) is a time-efficient method of training used worldwide. Its use has increased in the last few years among the population that seeks faster results and less time.^[8]^ The training programs are variable according to the objectives and characteristics of their practitioners, increasing their physical condition and improving body composition with the most outstanding benefits, according to experts and manufacturers.^[9–11]^ Previous studies^[12–15]^ verified the effectiveness of WB-EMS and its use as an alternative sports activity, both for those who flee from conventional methodologies and for athletes who want to improve their performance in sports through WB-EMS sessions. This training method has also shown improvements in body composition and strength in the elderly^[16–20]^ and in the active and healthy population.^[21,22]^

One of the hypotheses that can explain these alterations are, on the one hand, neural alterations, which occur first during a force training program, adaptations caused in the central nervous system that translate into an improvement in intra- and inter-muscular coordination that results in a greater capacity for muscle recruitment and therefore, greater capacity to produce force.^[23,24]^ On the other hand, the improvement in body composition can be explained by a higher energy expenditure caused by the intense and simultaneous work of large muscles and an increase in the post-session basal metabolic rate.^[25–27]^

To improve our knowledge on the use of WB-EMS and its effects on body composition and strength improvement, this meta-analysis aims to provide a systematic review of published studies that associate these two variables. The primary objective from this study is determine the efficacy of WB-EMS for the improvement of the composition and secondly evaluate the effects of this training on some strength parameters.

## 2 Methods

### 2.1 Study registration

This study has been registered in INPLASY (INPLASY202120050). It is reported in accordance with the Preferred Reporting Element Guidelines for Systematic Reviews and Meta-Analysis Protocol Statement.^[28]^

### 2.2 Eligibility criteria

#### 2.2.1 Study types

This study will include randomized clinical trials investigating the effects of whole-body electromyostimulation training on body composition and strength indicators

#### 2.2.2 Intervention types

In the intervention group, all subjects must have performed the same exercise protocol with the full-body electrostimulation suit. In the control group, participants must not have undergone any training program.

#### 2.2.3 Participant types

Those studies that include trained or untrained participants older than 18 years, without previous experience with WB-EMS will be considered.

#### 2.2.4 Outcome measurements

The primary outcomes of the study are fat-free mas or muscle mass and the percentage of fat mass or amount of fat measured by electrical bioimpedance, dual-energy X-ray absorptiometry, skinfolds, or anthropometric measurements.

The secondary results will be the maximum strength and muscle power measured in different tests.

### 2.3 Search strategy

Studies will be searched in the following electronic databases: PubMed, Web of Science, Scopus, SPORTDiscus and EMBASE until November 30 only in English language, without limitations of publication status. All randomized clinical trials will be considered. Details of the Cochrane Library are presented in Table 1.

**Table 1:** Search terms used in literature search.

Category 1	Category 2	Category 3
Whole body electro muscle stimulation WB-EMS Whole-body-electro-myo-stimulation Whole-body Electromyostimulation Whole-Body Electromyostimulation training Neuromuscular Electrical Stimulation NMES	Body Composition Fat Mass OR/AND Muscle Mass Strength OR/AND Power	Randomized controlled trial Controlled trial Clinical trial

### 2.4 Study Selection

Two authors will independently scan the studies through titles / abstracts; and unrelated studies will be excluded. Then, they will read the full articles of the remaining studies according to the eligibility criteria. The study selection process will be shown in a flow chart according to the Preferred Reporting Element Guidelines for Systematic Reviews and Meta-Analysis guidelines. If any divergence occurs, we will invite a third author to help resolve them.

### 2.5 Data collection and management

Two authors will independently collect data from all studies based on the data extraction form. It will consist of collecting the following information: title, authors, study design, characteristics of the participants, type of treatments and controls, results and other essential information elements. Any discrepancies will be discussed with an experienced third author through discussion.

### 2.6 Missing data dealing with

Once missing or unclear data is identified, we will contact the original trial authors to request it. Otherwise, we will analyse the available data if we cannot achieve it.

### 2.7 Risk of bias assessment

The risk of bias will be assessed by two independent researchers using the PEDro scale.^[29]^ In addition, the Grading of Recommendations Assessment, Development and Evaluation^[30]^ system will be used to classify the quality of the evidence and the strength of the recommendation.

### 2.8 Data synthesis

The *I*^2^ statistic will be used to assess heterogeneity. Also, we will make the Funnel Plot chart to assess the publication bias. If possible, we will do a meta-regression analysis and include both the random-effects and fixed-effects models.

### 2.9 Subgroup analysis

According to the different details of intervention, study quality and outcome indicators we will perform subgroup analysis.

### 2.10 Sensitivity analysis

We will do a sensitivity analysis to examine the robustness and stability of the synthesized results by eliminating low quality studies.

### 2.11 Reporting bias

Reporting bias will be detected using Funnel plot and Egger egression test if > 10 studies are included.

### 2.12 Ethics and dissemination

Ethical approval is not required as individual patient data will not be collected in this study. We will publish this study in a peer-reviewed journal.

## 3 Discussion

The whole body electromyostimulation has been shown to be effective in improving body composition in both pre-and postmenopausal women and in trained subjects.^[17,31]^ In addition, previous studies have also reported an increase in strength and power in the elderly,^[32]^ as well as in professional athletes.^[12,33–35]^ However, no systematic review evaluated its effects on both variables in all its practitioners. Therefore, this study is the first to systematically and comprehensively evaluate the effects of whole-body electrostimulation on body composition and strength in subjects over 18 years of age. The results of this study can provide evidence to determine whether WB-EMS can be effective in improving body composition (decreased fat and increased muscle mass) and strength and power levels.

With the results of this review, if the training method is effective in different populations for different objectives, we can use the methodology in a more specialized and effective way. More targeted programs will be created for altering body composition, either for the general population that does physical exercise to maintain their physical and mental health or for subjects who need to optimize their composition for health and quality of life issues, such as cases, of the elderly and of people with diseases such as osteopenia, osteoporosis, obesity, among others.

On the other hand, if WB-EMS is effective in terms of strength parameters, adapted programs will be applied to improve this ability in athletes to increase performance or in the general population to maintain physical condition and improve fitness. quality of life.

## 4 Limitations

The criteria used to judge the level of evidence have not yet been standardized. Different authors of systematic reviews use different criteria, and the same author may use different criteria in different studies.^[36]^ The use of different criteria is related to the decision to include only randomized clinical trials or to also consider studies of low methodological quality, in which the measurement scales may also vary.^[37]^ Furthermore, the best method of assessing risk of bias has not been determined. The search strategy only searched for articles in English, which implied a risk of bias since publishing significant results is easier than publishing non-significant results, and the latter are more likely to appear in national journals written in languages other than English.^[38]^

## 5 Review status

The team at this time is waiting to have the systematic review protocol published to begin the first step of the review. This review is expected to be complete in April 2021.

## Author contributions

RLS and AJC were the initiator of the Project. RSL, AJC, HL PGJ and CVJ were responsible for writing this protocol. HMMA and RLS contributed to the idea development and the development of the Project. All authors have read and approved the protocol.

**Conceptualization:** Luiz Rodrigues-Santana, Jose Carmelo Adsuar, Jorge Carlos-Vivas.

**Writing – original draft:** Luiz Rodrigues-Santana, Jose Carmelo Adsuar, Hugo Louro, Jorge Pérez-Gómez, Miguel Angel Hernández-Mocholí, Jorge Carlos-Vivas, Rossana Gómez Campos, Luis Felipe Castelli Correia de Campos.

**Writing – review & editing:** Luiz Rodrigues-Santana, Hugo Louro, Jorge Pérez-Gómez, Miguel Angel Hernández-Mocholí, Jorge Carlos-Vivas, Rossana Gómez Campos, Luis Felipe Castelli Correia de Campos.
